# A synonymous codon variant in two patients with autosomal recessive bestrophinopathy alters in vitro splicing of *BEST1*

**Published:** 2010-12-31

**Authors:** Alice E. Davidson, Panagiotis I. Sergouniotis, Rosemary Burgess-Mullan, Nichola Hart-Holden, Sancy Low, Paul J. Foster, Forbes D.C. Manson, Graeme C.M. Black, Andrew R. Webster

**Affiliations:** 1 School of Biomedicine, The University of Manchester, Manchester Academic Health Science Centre, Central Manchester University Hospitals NHS Foundation Trust, Manchester, UK; 2 Moorfields Eye Hospital, London, UK; 3 Institute of Ophthalmology, University College London, London, UK

## Abstract

**Purpose:**

Autosomal recessive bestrophinopathy (ARB) is a newly defined retinal dystrophy caused by biallelic mutations in bestrophin-1 (*BEST1*) and is hypothesized to represent the null bestrophin-1 phenotype in humans. The aim was to determine whether a synonymous *BEST1* variant, c.102C>T, identified in two unrelated ARB patients, alters pre-mRNA splicing of the gene. Additionally a detailed phenotypic characterization of this distinctive condition is presented for both patients.

**Methods:**

*BEST1* was analyzed by direct sequencing. Patients underwent standard ophthalmic assessment. In silico and in vitro analysis using a minigene system was performed to assess whether a synonymous variant identified, c.102C>T p.Gly34Gly, alters pre-mRNA splicing of *BEST1*.

**Results:**

Both ARB patients harbored either proven (patient 1; c.102C>T p.Gly34Gly and c.572T>C p.Leu191Pro) or presumed (patient 2; c.102C>T p.Gly34Gly and c.1470_1471delCA, p.His490GlnfsX24) biallelic mutations in *BEST1* and were found to have phenotypes consistent with ARB. In vitro analysis of the synonymous variant, c.102C>T p.Gly34Gly, demonstrated it to introduce a cryptic splice donor site 52 nucleotides upstream of the actual splice donor site.

**Conclusions:**

The novel *BEST1* variant identified, c.102C>T p.Gly34Gly, alters pre-mRNA splicing in vitro and is potentially pathogenic. In vivo this splicing variant is predicted to lead to the production of an mRNA transcript with a premature termination codon (p.Glu35TrpfsX11) that is predicted to be degraded by NMD.

## Introduction

Autosomal recessive bestrophinopathy (ARB) is caused by biallelic mutations in *BEST1* [1]. *BEST1* encodes bestrophin-1, a transmembrane protein primarily expressed in the basolateral membrane of the retinal pigmented epithelium (RPE) [2]. Best vitelliform macular dystrophy (BVMD) [3] and autosomal dominant vitreoretinochoroidopathy (ADVIRC) [4] are also associated with mutations in *BEST1*. Although the functional role of bestrophin-1 within the RPE remains uncertain, with postulated functions as a Ca^2+^ activated Cl^-^ channel [5], a regulator of voltage gated Ca^2+^ channels [6], or as a HCO_3_^–^ channel [7] the study of disease-associated *BEST1* variants has helped to elucidate pathogenic mechanisms underlying the bestrophinopathies. BMVD [3] and ADVRIC [4] are both hypothesized to arise from gain-of-function mutations that exert a dominant negative effect of the wild-type allele, whereas ARB is hypothesized to result from biallelic functionally null mutations and thus represents the null bestrophin-1 phenotype in humans [1,8,9].

Ten compound heterozygous or homozygous mutations have been identified in seven families diagnosed with ARB [1,8,9]. Affected individuals present with central vision loss, abnormal dark and light adapted full-field electroretinograms (ERGs) and a severely reduced electro-oculogram (EOG) light-rise that cannot be explained by the magnitude of the ERG abnormalities. On fundoscopy, widespread RPE irregularity and small, pale subretinal deposits, more clearly demonstrated on autofluorescence (AF) imaging, are observed. *BEST1* expression is higher in the peripheral RPE compared to the macular RPE [10]. A lack of bestrophin-1 across the entire RPE (null phenotype) may explain the more widespread and progressive photoreceptor dysfunction and the widespread punctuate flecks observed in the peripheral retina in patients with ARB.

In this report we investigate how a synonymous *BEST1* variant, identified in two unrelated patients with a clinical diagnosis of ARB, affects pre-mRNA splicing, by performing an ex vivo splice assay. The clinical phenotype is presented, further establishing ARB as a distinctive bestrophinopathy.

## Methods

### Study subjects and clinical examination

Two unrelated patients with a diagnosis of ARB were identified in Moorfields Eye Hospital, London, UK. After informed consent was obtained, blood samples were donated and genomic DNA was extracted from peripheral blood lymphocytes. The study was approved by the Moorfields and Whittington Hospitals’ local ethics committee.

Clinical assessment included: full medical history, best-corrected Snellen visual acuity, dilated fundus examination, color fundus photography, AF with a confocal scanning laser ophthalmoscope (cSLO; HRA 2) and spectral-domain optical coherence tomography (SD-OCT; Spectralis). EOG, ERG, and pattern ERG procedures were performed according to the International Society for Clinical Electrophysiology of Vision (ISCEV) Standards [11-13]. Both patients were examined more than twice over at least five years, making a longitudinal evaluation of the phenotype possible.

### DNA sequencing

All ten coding exons and flanking intronic boundaries of *BEST1* were analyzed by direct sequencing from PCR amplicons [1]. The absence of putative *BEST1* mutations was confirmed by single-stranded conformation polymorphisms (SSCP) in 210 white European control chromosomes [1]. The cDNA is numbered according to Ensembl transcript ID ENST00000378043.

### Ex vivo splice assay cloning

Due to the limited expression pattern of *BEST1* we could not evaluate the effect of c.102C>T on splicing in patient-derived RNA. We therefore used an alternative ex vivo splice assay approach. A plasmid encoding a wild-type *BEST1* fragment was generated by PCR amplification from genomic DNA. The fragment was sub-cloned into the α-globin–fibronectin–extra domain B (EDB) minigene [14]. The c.102C>T, p.Gly34Gly variant was introduced into the wild-type construct by site-directed mutagenesis using the QuickChange II Kit (Stratagene, Cheshire, UK) in accordance with the manufacture’s protocol. All constructs generated were sequenced to ensure fidelity and orientation.

### Ex vivo splice assay

Wild-type and mutant (c.102C>T, p.Gly34Gly) EDB minigene constructs were transiently transfected into HEK293 cells using Lipofectamine reagent (Invitrogen, Paisley, UK). After 24 h, the cells were pelleted and RNA was extracted using a QIA shredder kit (Qiagen, Crawley,  UK) and an RNeasy mini kit (Qiagen) according to the manufacturer’s instructions. After DNase treatment (Promega, Hampshire, UK), cDNA was produced by reverse transcription (RT) PCR from approximately 1 μg RNA. Vector-specific primers were used to establish cDNA linearity loading controls for the experimental PCR assays.

## Results

### Clinical findings

**Figure 1:**
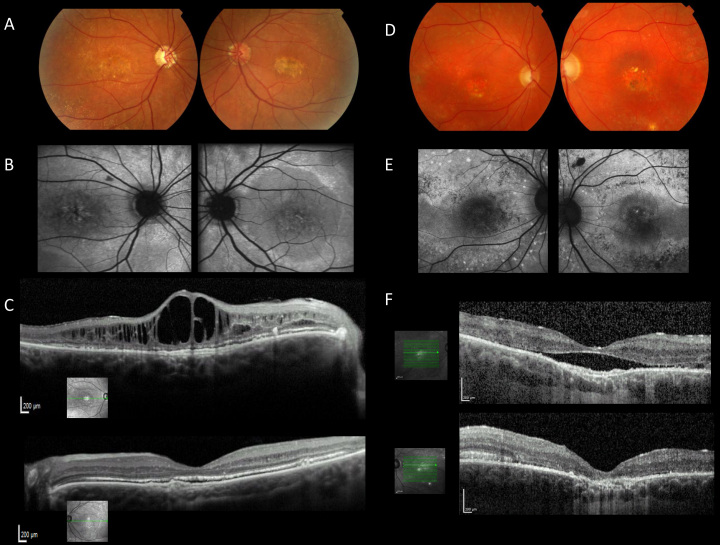
Color fundus photographs showing fundus autofluorescence (AF) imaging and horizontal spectral domain optical coherence tomography (OCT) scans of affected individuals. **A**–**C**: patient 1 at 44 years of age; **D**–**F**: patient 2 at 45 years of age. Fundus pictures show widespread retinal pigment epithelium (RPE) alterations and yellowish subretinal deposits along the vascular arcades as well as yellow-white material in the maculae (**A**, **D**). Changes are more visible on AF imaging as diffuse, discrete areas of hyper and hypoautofluorescence (**B**, **E**). On OCT, intraretinal or subretinal fluids as well as atrophy are shown (**C**, **F**).

Subject 1, a 44-year-old female of white European origin was diagnosed initially with macular dystrophy at the age of 19 years. No family history of retinal problems was reported. She was first noted to have poor central vision in a routine eye test at the age of nine. The finding was predominant in the left eye and a diagnosis of amblyopia was made. Bilateral YAG laser iridotomies were performed at the age of 35 years, followed by trabeculectomy and vitrectomy for malignant glaucoma on the right eye. Pattern ERG performed at the age of 41 was undetectable on the right and within normal limits on the left. Generalized retinal dysfunction affecting rod more than cone photoreceptors on full-field ERGs was recorded, and evidence of additional dysfunction affecting the photoreceptor/RPE interface with a severely subnormal EOG light rise bilaterally was observed. Humphrey field testing at age 41 demonstrated significant field loss in the right eye. When examined at age 44, best-corrected visual acuities were 1.0 LogMAR in the right and 0.8 LogMAR in the left eye with a hyperopic correction (+3 D) on the right eye. The patient had glaucoma and was being treated with systemic acetazolamide and topical treatment for glaucoma, with intraocular pressures being controlled. Anterior segment OCT imaging showed angle closure in both eyes. Fundoscopy showed chronic cystoid macular edema on the right and pale confluent deposits in the fovea and midperiphery of both eyes. Interestingly, bilateral nasal juxtapapillary drusen were observed (Figure 1A) and the patient was therefore screened and excluded for the c.245C>T p.Arg345Trp mutation in *EFEMP1*. Fundus photographs, autofluorescence imaging and spectral domain OCTs are shown in Figure 1A-C.

Subject 2, a 45-year-old white European male, was diagnosed initially with Stargardt macular dystrophy at 11 years and with narrow angle glaucoma at 21. The patient has no family history of ocular disease. His two young children have been reported to have normal vision. Problems with central vision were first noted in early childhood and gradual deterioration over the years was reported. Bilateral YAG laser iridotomies were performed at the age of 22. Electrophysiology performed at the age of 40 showed only residual activity in pattern ERG. Full-field ERGs were in keeping with generalized retinal dysfunction involving the cone and rod systems. When examined at age 44, best-corrected visual acuities were 1.0 LogMAR for the right and 0.8 LogMAR for the left eye. IOPs were controlled with systemic and topical treatment for glaucoma. Fundoscopy showed atrophic lesions in both maculae with small yellowish subretinal deposits in the fovea and around the vascular arcades (Figure 1D). The changes are better visible on autofluorescence imaging (Figure 1E). Spectral domain OCT showed subretinal fluid between RPE and neurosensory retina in the right and atrophic changes in the left macula (Figure 1F). Clinical details of subjects 1 and 2 are summarized in Table 1.

**Table 1:** Clinical Details of Two Individuals Affected with Autosomal Recessive Bestrophinopathy.

**Individual lD**	**Gender**	**Age**	**Visual Acuity (LogMAR equivalent)**	**Age at diagnosis (initial diagnosis)**	**Angle closure glaucoma**	**Retina**	**ERG**	**EOG**	**Mutations: cDNA (protein)**
Subject 1	Female	44	OD 1.0 LogMAR OS 0.8 LogMAR	19 (macular dystrophy)	Yes	macular edema on the right eye; pale deposits in the fovea and midperiphery of both eyes	Pattern ERG undetectable on the right, normal on the left; subnormal rod ERGs and bright flash ERG a-waves bilaterally; delayed and subnormal 30Hz flicker ERGs bilaterally.	Markedly subnormal EOG light rise bilaterally	c.102C>T (p.Gly34Gly) c.572T>C (p.Leu191Pro)
Subject 2	Male	45	OD 1.0 LogMAR OS 0.8 LogMAR	11 (macular dystrophy)	Yes	atrophic lesions in both maculae; pale deposits in the fovea and around the vascular arcades of both eyes	Pattern ERG undetectable bilaterally; subnormal rod ERGs and bright flash ERG a-waves bilaterally; delayed and subnormal 30Hz flicker ERGs bilaterally.	Not presented	c.102C>T (p.Gly34Gly) c.1470_1471delCA (p.His490GlnfsX24)

Abbreviations: OD represents right eye; OS represents left eye, ERG represents electroretinogram, EOG represents electro-oculogram.

### Molecular findings and in silico analysis

Sequencing all the coding exons of *BEST1* in patient 1 identified two novel heterozygous variants: c.102C>T p.Gly34Gly and c.572T>C p.Leu191Pro. The proband’s asymptomatic son was subsequently found to harbor only the latter variant, confirming that the changes were in trans. A multiple alignment of bestrophin-1 shows that the leucine residue at position 191 is highly conserved down to *Danio rerio* (data not shown). Patient 2 was found to have compound heterozygous mutations, comprising both the previously reported frame-shift mutation, c.1470_1471delCA, p.His490GlnfsX24 [15] and the novel variant, c.102C>T, p.Gly34Gly. The novel *BEST1* variants (c.102C>T p.Gly34Gly and c.572T>C p.Leu191Pro) were absent in 210 white European control chromosomes tested.

To predict whether the c.102C>T p.G34G variant affects exonic splice regulatory sequences and/or generates a cryptic splice site within *BEST1,* the wild-type and mutated sequences of exon 2 were analyzed using pre-mRNA splicing prediction programs. The RESCUEese website [16] predicts that no exonic splice enhancer (ESE) sites are present in either the wild-type or mutant sequence. The PeSX website [17,18] predicts that the wild-type sequence contains an ESE which is abolished in the mutant sequence. The ESE finder website [19] predicts that the mutation abolishes an SRp55 binding site present in the wild-type sequence. The FAS-ESS website [20] predicts that no exonic splice silencers (ESS) sites are present in either the wild-type or mutant sequence. Splice site prediction tools, Human Splicing Finder (HSF) [21], NNSPLICE [22] and NetGene2 [23] all predict that the variant may create a cryptic splice donor site 52 nucleotides upstream of the genuine splice donor site.

### Ex vivo α-globin-fibronectin-EDB splice assay

To test whether the *BEST1* variant c.102C>T affects pre-mRNA splicing, an ex vivo splice assay was performed. Wild-type and mutant (c.102C>T p.Gly34Gly) sequences of *BEST1* exon 2 and the surrounding intronic regions were cloned into the α-globin-fibronectin-EDB splice assay vector and transfected into HEK 293 cells. Assays were performed as previously described [14].

**Figure 2:**
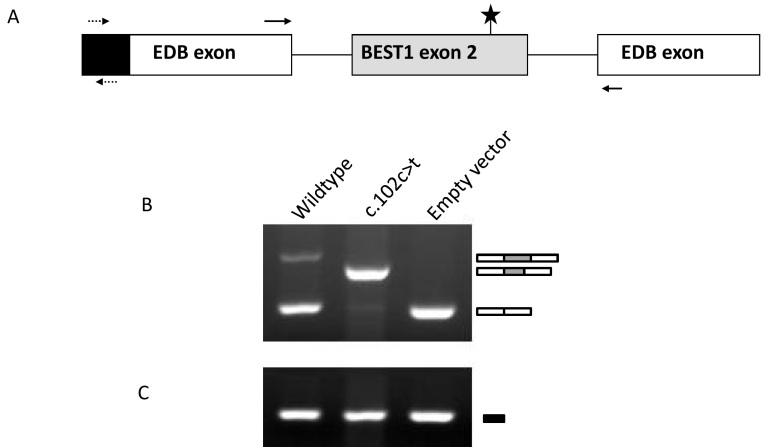
The ex vivo splicing assay. **A**: Schematic representation of the α-globin-fibronectin- extra domain B (EDB) splice assay construct. Wild-type and mutant (c.102C>T) forms of *BEST1* exon 2 with flanking intronic sequence were cloned into the α-globin-fibronectin-EDB splice assay vector. The position of the mutated residue is highlighted with a star, and primer binding sites to exonic vector sequences are indicated with arrows. **B**: Splicing products generated by RT–PCR were separated by agarose gel electrophoresis as indicated. The identity of the spliced products was established by direct sequencing and is schematically represented on the right. **C**: Agarose gel of RT–PCR reactions performed with control primers designed against the vector sequence (dashed arrows in **A**) demonstrates equal loading of the cDNA template. The figure represents results obtained from three separate experiments.

Analysis of the resulting splice products demonstrated that the wild-type and mutant constructs produced differently-spliced products (Figure 2). The wild-type construct was spliced to produce two products at approximately 480 bp and 250 bp, corresponding respectively to the vector exons spliced to *BEST1* exon 2, and the vector exons alone. It is notable that wild-type exon 2 is only weakly spliced. This result can most likely be attributed to the fact that we are not studying the exon within its native genomic context, a disadvantage of all in vitro splice assays. By contrast, the mutant construct was spliced to produce two products at approximately 430 bp and 250 bp, corresponding respectively to the vector exons spliced to a truncated version of *BEST1* exon 2 and the vector exons alone. The mutant product appeared to be spliced more efficiently than the wild-type product, suggesting that the cryptic splice site introduced by the variant had a very strong effect on splicing in this system. The identity of all alternatively spliced products was established by direct sequencing and demonstrated that the c.102C>T variant creates a cryptic splice donor site 52 nucleotides upstream of the genuine splice donor site, supporting the predictions made by the splice prediction tools HSF, NNSPLICE, and NetGene2 [21-23].

## Discussion

Since the first description of ARB as a novel retinal dystrophy caused by bialleic mutations in *BEST1*, our understanding of the clinical presentation and pathophysiology of the condition has progressed [1,8,9]. The two unrelated probands reported here both displayed key clinical features of the condition, including loss of central vision in early in life, angle-closure glaucoma, subretinal and intraretinal fluid accumulation, a lack of a dominant mode of inheritance, and abnormal electrophysiology (ERG and EOG light rise). Both patients presented with recessive macular dystrophy in their second decade of life and later developed glaucoma. This finding is concordant with our previous study in which all ARB patients described were found to be hyperopic and 3/7 patients were also diagnosed with angle-closure glaucoma [1]. Importantly for both probands in this study, the angle-closure glaucoma contributed to visual loss, and we therefore recommend that all ARB patients be routinely screened for angle-closure disease and associated glaucoma once diagnosed with the condition. *BEST1*’s role within ocular development is poorly understood. In the light of our current finding that ARB is frequently associated with angle-closure glaucoma and that *BEST1* mutations cause the developmental ocular disorder ADVIRC [4], we believe the role of *BEST1* in ocular development and glaucoma merits further investigation.

Patient 1 was found to have one previously reported missense change and one novel synonymous variant in *BEST1* (c.102C>T p.Gly34Gly and c.572T>C, p.Leu191Pro). The proband’s asymptomatic son was subsequently found to harbor only the latter variant, confirming that the changes were in trans. Patient 2 had one frame-shift mutation and the same synonymous variant in *BEST1* as patient 1 (c.1470_1471delCA p.His490GlnfsX24 and c.102C>T p.Gly34Gly). It was not possible to determine if the mutations identified in patient 2 were in cis or trans as no familial DNA samples were available for segregation analysis. However, the phenotypic presentation of patient 2 is in keeping with ARB and not BVMD. As ARB is caused by biallelic mutations in *BEST1* [1], a second pathogenic variant in trans to the c.1470_1471delCA variant was likely. The in silico and in vitro data presented support the belief that the c.102C>T p.Gly34Gly variant is likely to be pathogenic, and hence the second disease causing allele in the patient. We therefore hypothesize, based on the circumstantial evidence presented, that the c.102C>T variant identified in patient 2 is pathogenic and in trans to c.1470_1471delCA. A more parsimonious, but not impossible interpretation of these data are that the c.102C>T p.Gly34Gly variant is benign, has by chance only ever been found in these two phenotypically similar patients and that both have unusual manifestations of dominant disease.

We hypothesized that the c.102C>T p.Gly34Gly variant, located at the 3′ end of the first translated exon of *BEST1*, is pathogenic by altering the pre-mRNA splicing. An in vitro splice assay demonstrated the introduction of a cryptic splice donor site 52 nucleotides upstream of the actual splice donor site. Therefore, in vivo the synonymous variant is predicted to lead to the production of an mRNA transcript with a premature stop codon (p.Glu35TrpfsX11) that would be presumed to be degraded by nonsense-mediated decay (NMD). Previously we have suggested that ARB represents the null bestophin-1 phenotype in humans, as patients with biallelic null mutations have similar phenotypic characteristics to patients with compound heterozygous missense mutations in *BEST1*. The mutations identified here in two further patients support this proposition. In patient 2 both variants, which we presume to be in *trans,* produce transcripts that are predicated to be degraded by NMD; p.His490GlnfsX24 and p.Glu35TrpfsX11. Patient 1 has a similar phenotype to patient 2 and other ARB patients. This suggests that the missense isoform in patient 1 lacks sufficient function, and that in conjunction with the second allele transcript that is predicted to be degraded by NMD, the patient is functionally null for bestrophin-1.

Examples of synonymous exonic mutations introducing cryptic splice donor sites have previously been reported [24,25]. However the potential for such synonymous codon changes to have pathogenic consequences are often overlooked due to the strong association of exonic mutations with solely protein coding changes. The work presented here demonstrates the power of combining detailed phenotypic analysis with comprehensive in silico and in vitro analysis of a synonymous variant to facilitate an informed and accurate diagnosis. With the ever-advancing pace of high throughput DNA sequencing technologies, determining the relevance of such synonymous variants is becoming increasingly important.
